# Authors’ Reply: Bridging Neurofeedback and Structural Connectivity in Chronic Pain

**DOI:** 10.2196/89007

**Published:** 2026-01-28

**Authors:** Luke Spencer Bialostocki, Divya Bharatkumar Adhia, Damith Rathnayake Mudiyanselage, Mark Llewellyn Smith, Yusuf Ozgur Cakmak, Dirk De Ridder, Ramakrishnan Mani, Jerin Mathew

**Affiliations:** 1 Department of Anatomy, Faculty of Biomedical Sciences, University of Otago, 270 Great King Street, Dunedin, Otago, 9016, New Zealand, 64 035565036; 2 Department of Surgical Sciences, Faculty of Medicine, University of Otago, Dunedin, Otago, New Zealand; 3 Neurofeedback Services of New York, New York, NY, United States; 4 Centre for Health, Activity, and Rehabilitation Research, School of Physiotherapy, University of Otago, Dunedin, Otago, New Zealand

**Keywords:** brain-computer interface, brain oscillations, chronic pain, electroencephalography, musculoskeletal, neuromodulation

We appreciate the opportunity to respond to the letter [1] written in response to our recent publication “Neurofeedback Training for Managing Neuropathic Pain–Like Features in Chronic Musculoskeletal Pain: Protocol for an Open-Label Pilot Feasibility Clinical Trial” [2]. The paper outlines a protocol for a novel electroencephalography-based neurofeedback approach, which aims to simultaneously downregulate infraslow activity in the right insula and the dorsal anterior cingulate cortex.

We thank the authors for their interest in our publication and for suggesting the incorporation of diffusion tensor imaging (DTI) to assess neuroplastic alterations in the cingulum bundle. We agree that DTI could provide valuable complementary information on the structural substrates underlying functional changes induced by electroencephalography-based neurofeedback. While we recognize the merit of this proposal, particularly the possibility that structural differences could influence training outcomes, we are unfortunately unable to incorporate DTI into the present study. Access to DTI is not available within the scope or resources of this project, as medical imaging technologies such as DTI are costly to implement and limited to specialized centers with the necessary equipment and trained personnel. In addition, our study has now completed its recruitment and data collection phase. For these reasons, integrating DTI into this particular study is not feasible.

Structural imaging techniques such as DTI may indeed be incorporated into future studies to further elucidate the neurophysiological changes associated with endogenous neuromodulation. These suggestions underscore a promising direction for integrating neuroanatomical perspectives with neuromodulatory approaches, offering a more complete understanding of the anatomical processes involved. Integrating DTI also represents a promising direction for future research aiming to link functional neuromodulation with white matter pathways [3]. However, it is also important to note that structural and functional connectivity often show weak correlations [4].

## Abbreviations

DTI: diffusion tensor imaging
